# Chemo-Enzymatic Synthesis of Optically Active γ- and δ-Decalactones and Their Effect on Aphid Probing, Feeding and Settling Behavior

**DOI:** 10.1371/journal.pone.0146160

**Published:** 2016-01-07

**Authors:** Filip Boratyński, Katarzyna Dancewicz, Marlena Paprocka, Beata Gabryś, Czesław Wawrzeńczyk

**Affiliations:** 1 Department of Chemistry, Wrocław University of Environmental and Life Sciences, Wrocław, Poland; 2 Department of Biology and Ecology, University of Zielona Góra, Zielona Góra, Poland; University of Basilicata, ITALY

## Abstract

The enantiomerically enriched γ- and δ-decalactones (**4a** and **4b**) were prepared from corresponding racemic primary-secondary 1,4- and 1,5-diols (**1a** and **1b**), as products of enzymatic oxidation catalyzed by different alcohol dehydrogenases. The results of biotransformations indicated that the oxidation processes catalyzed by alcohol dehydrogenase (HLADH), both isolated from horse liver and recombinant in *Escherichia coli*, were characterized by the highest degree of conversion with moderate enantioselectivity of the reaction. Useful, environmentally friendly extraction procedure of decalactones (**4a** and **4b**) based on hydrodistillation using a Deryng apparatus was developed. Both racemic lactones (**4a** and **4b**), as well as their enantiomerically enriched isomers, were tested for feeding deterrent activity against *Myzus persicae*. The effect of these compounds on probing, feeding and settling behavior of *M*. *persicae* was studied *in vivo*. The deterrent activity of decalactones (**4a** and **4b**) against aphids depended on the size of the lactone ring and the enantiomeric purity of the compounds. δ-Decalactone (**4b**) appeared inactive against *M*. *persicae* while γ-decalactone (**4a**) restrained aphid probing at ingestional phase. Only (–)-(S)-γ-decalactone (**4a**) had strong and durable (i.e. lasting for at least 24 hours) limiting effect, expressed at phloem level.

## Introduction

Compounds with the lactone moiety, regarding to their broad range of valuable properties, have been the subject of many studies. Among different biological activities, the most widely known is antimicrobial activity [1, 2]. They also exhibit strong cytostatic activity. Compounds with the lactone structure on the one hand occur as antifeedants [3–7], on the other play pheromones role in the world of insects [8]. Lactones are described in the literature as important flavor compounds [9–12]. They exist as naturally occurring odorants in all major food groups and in many different beverages.

The odoriferous attributes of γ- and δ-decalactones (**4a** and **4b**), which are widely spread in the nature, are well documented [10, 13]. Privileged, occurring predominantly natural form of such lactones are the (*R*)-enantiomers, which are responsible for a pleasant, naturally fruity, aromatic note. Their odor is more intense compared to the (*S*)-isomers. (+)-(*R*)-γ-Decalactone (**4a**) exhibits intense, fruity with peach note odor, in opposite to (–)-(*S*)-γ-enantiomer (**4a**), which aroma is characterized as less intense, sweet, coconut with fruity note. γ-Decalactone (**4a**) is used in the formulation of fruit aromas such as strawberry and peach. Besides, it is also present in various fermented products such as bread and whisky [14]. (+)-(*R*)-δ-Decalactone (**4b**), which is sweet, creamy and has a nut-like odor, was identified in fat-containing food, such as cheese and butter. (+)-(*R*)-δ-Decalactone (**4b**), already used as food additive, is mixed with margarine to give a flavor similar to that of butter. Apart from the food industry it is used as a fragrance composition in the cosmetics industry and a flavor in the tobacco industry. Both lactones with their tempting aroma and low odour thresholds are currently one of the main products derived from aroma biotechnology.

γ- and δ-Decalactones (**4a** and **4b**) have been widely investigated with regard to their flavor, but only few studies have been conducted on their other biological activities. Nevertheless, biological studies point at the very broad spectrum of acivities of γ- and δ-decalactones (**4a** and **4b**). For example, it has been shown that both decalactones (**4a** and **4b**) inhibit the growth of selected filamentous fungi (*Aspergillus niger*), yeast (*Candida albicans*) and bacteria (*Staphylococcus aureus*) [15]. δ-Decalactone (**4b**) has been detected in venom-producing mandibular glands of ponerine ant *Pachycondyla apicalis* [16] and (+)-(*R*)-δ-decalactone (**4b**) was reported as a component of the warning odor of North American porcupine *Erethizon dorsatum* [17]. γ-Decalactone (**4a**) as a component of the sex pheromone of scarab beetles *Osmoderma eremita* plays an important role in the communication process between insects [18–20]. It has been proven that only the (+)-(*R*)-γ-decalactone (**4a**) is active in relation to the female beetles being responsible for the characteristic fruity, peach and plum scent secreted by the male beetles. At the same time, (+)-(*R*)-γ-decalactone (**4a**) is used by the larval predator, the click beetle *Elater ferrugineus*, as a kairomone to locate *O*. *eremita*, which is its prey [20].

Biological properties of chiral compounds are often strongly related to the absolute configuration [21]. Therefore, looking for new stereoselective methods of synthesis of the optically pure molecules with various biological activities is one of the most developed areas in current chemistry. One of the method from green chemistry toolbox is biotransformation showing overwhelming advantages over synthetic chemistry approach. Synthesis involving enzymes allows stereochemical control of reactions and usually leads to optically active compounds, in the ideal case, enantiomerically pure. Biotransformations are characterized by high enantio-, regio- and chemoselectivity. Enzymes, through a detailed diagnosis of the three-dimensional structure of the compound by the enzymatic pocket, have high substrate specificity. It is also possible to provide reactions of low-reactive chemicals and reactions of not activated areas of the molecule. This often leads to receive products impossible to obtain by chemical synthesis. The ability to conduct biotransformation under mild conditions (temp, pH) reduces the number of possible side reactions. Moreover, biocatalysis decreases the consumption of chemicals, which is associated with a significantly smaller amount of toxic chemical waste.

Among different biocatalytic strategies of the synthesis of lactones [22–25], we have applied the enzymatic oxidation of aliphatic diols [26]. Encouraged by our latest valuable results [27], concerning biosynthesis of *cis*- and *trans*-whisky lactones, we have focused on enzymatic oxidation of long chain primary-secondary diols. In our current investigations, we have applied eight commercially available alcohol dehydrogenases in the stereoselective oxidation of 1,4- and 1,5-decadiols (**1a** and **1b**) to the corresponding γ- and δ-decalactones (**4a** and **4b**).

Taking into consideration the aforementioned broad spectrum of lactone properties it was purposeful to check whether γ- and δ-decalactones (**4a** and **4b**), besides their documented odoriferous, antimicrobial and attractant properties indicate also antifeedant activity. Our interests in this area is inspired by literature data describing the natural and synthetic compounds with γ- and δ-lactone function that are known as active feeding deterrents against many insects, including aphids [3–6]. In regard of this, both racemic γ- and δ-decalactones (**4a** and **4b**), as well as their enantiomerically enriched isomers, were studied for feeding deterrent activity against the polyphagous peach potato aphid *Myzus persicae* (Sulz.).

## Materials and Methods

### Analysis

The purity of synthesized products was checked by thin layer chromatography (TLC), using aluminum foil plates coated with silica gel. Compounds were detected by spraying the plates with 1% Ce(SO_4_)_2_ and 2% H_3_[P(Mo_3_O_10_)_4_] in 10% H_2_SO_4_. The crude products were purified by preparative column chromatography using silica gel (Kieselgel 60, 230–400 mesh, Merck) with mixtures of hexane-acetone (in various ratios) as eluents.

The progress of reaction and enantiomeric excesses of the lactones were determined by gas chromatography (GC). Gas chromatography analyses were performed on Agilent Technologies 6890N and Varian Chrompack CP-3380 instruments, using HP-20M-Carbowax (cross linked phenyl methyl siloxane) capillary column (25 m x 0.32 mm x 0.3 μm); DB-17 (cross linked phenyl methyl siloxane) capillary column (30 m x 0.25 mm x 0.25 μm). Chiral column: CP7502 Chirasil-dex CB (25 m x 0.25 mm x 0.25 μm) were used to the enantiomeric excesses determination.

The structures of the compounds were determined on the basis of ^1^H NMR, ^13^C NMR, Dept-135, HMQC, COSY and IR spectral data. ^1^H NMR spectra were recorded in CDCl_3_ solutions on Bruker Avance DRX 600 (600 MHz) spectrometer. IR spectra were determined using FTIR Thermo-Mattson IR 300 Spectrometer. Optical rotations were measured on an Autopol IV automatic polarimeter (Rudolph). The pH measurements were conducted on a HI 9321 Microprocesor pH Meter equipped with a glass electrode.

### Chemicals

Racemic γ- and δ-decalactones (**4a** and **4b**) were purchased from Sigma-Aldrich.

### Enzymes and coenzymes

The following alcohol dehydrogenases were used: Horse Liver Alcohol Dehydrogenase (HLADH) from Sigma-Aldrich; Yeast Alcohol Dehydrogenase (YADH) from Sigma-Aldrich; Horse Liver Alcohol Dehydrogenase recombinant from *Escherichia coli* (HLADH-rec) from Evocatal GMBH; *Lactobacillus Kefir* Alcohol Dehydrogenase (LKADH) from Fluka; Primary Alcohol Dehydrogenase screening kit (PADH I, II and III) from Codexis. *Bacillus Stearothermophilus* Alcohol Dehydrogenase (BSADH) was a generous gift from Protein Biochemistry Institute, Naples, Italy.

Coenzymes: nicotinamide adenine dinucleotide (NAD^+^), flavin mononucleotide (FMN) were purchased from Sigma-Aldrich, while nicotinamide adenine dinucleotide phosphate (NADP^+^) were purchased from Codexis.

### Synthesis of substrates for enzymatic oxidation

Decane-1,4-diol (**1a**) and decane-1,5-diol (**1b**) were prepared by the reduction of commercially available corresponding racemic γ- and δ-decalactones (**4a** and **4b**) with LiAlH_4_, according to the method described by us earlier [26]

### Enzymatic oxidation of racemic decane-1,4-diol (1a) and decane-1,5-diol (1b)

#### Screening procedure

In the screening experiments diol (**1a-b**) (0.02 g) and coenzymes: NAD(P)^+^ (0.007 g) and FMN (0.2 g) were dissolved in 0.1 M glycine-NaOH buffer (15 mL) at different value of pH (7.2, 8.5, 9.0) and temperature (25, 35, 45°C). The pH of the mixture was readjusted with 2 M NaOH. Then, the enzyme solution (0.002 g) dissolved in buffer (2 mL) was added dropwise. To determine the stability of the substrate the control sample, containing a buffer with the substrate without the addition of the enzyme, was conducted. Changes in pH during the biotransformation was monitored using a pH-meter. Samples (1 mL) of the reaction mixtures were taken after several time intervals (2, 24, 48 and 120 hours). The aqueous phase was acidified to pH = 3, washed with brine, then extracted with chloroform (1 mL). The extract was dried over anhydrous magnesium sulfate and analyzed by GC.

#### Preparative procedure

The preparative-scale transformations were performed fivefold in the same conditions as screening experiments using 0.1 g of diol (**1a-b**) (5 x 0.02 g), 0.035 g of NAD(P)^+^ (5 x 0.007 g), 1 g FMN (5 x 0.2 g) and 0.01 g enzyme (5 x 0.002 g). When the reaction was completed the reaction mixture was extracted by hydrodistillation method using a Deryng apparatus. The yields and optical rotations of prepared products are given below. The precise spectral data for prepared diastereoisomeric mixture of hemiacetals, which are in agreement with literature data [28], were also presented.

Oxidation of (**±**)**-1a** (0.1 g) by HLADH, after 48 hours, gave a mixture of: (–)-(*S*)-**4a** (79%) and mixture of hemiacetals **2a** and **3a** (21%). The column chromatography of this mixture afforded 0.063 g (63% yield) of (–)-(*S*)-**4a**, ee = 20%, [α]_D_^20^ = – 5.8° (*c* 1.5, CHCl_3_) and 0.011 g (11%) of hemiacetals **2a** and **3a**.

Oxidation of (**±**)**-1a** (0.1 g) by PADH III, after 120 hours, gave a mixture of the following products: unreacted (**±**)**-1a** (66%), (+)-(*R*)-**4a** (16%) and mixture of hemiacetals **2a** and **3a** (18%). The column chromatography of this mixture afforded 0.016 g (16% yield) of (+)-(*R*)-**4a**, ee = 80%, [α]_D_^20^ = + 17.6° (*c* 0.6, CHCl_3_) and 0.006 g (6%) of hemiacetals **2a** and **3a**.

### 5-Hexyltetrahydrofuran-2-ols (mixture of isomers 2a and 3a)

Colorless liquid, ${\text{n}}_{\text{D}}^{20}$ = 1.4322; *cis* (**2a**) and *trans* (**3a**) isomers of the composition 55: 45%; ^**1**^**H NMR** (600 MHz, CDCl_3_) δ: 0.88 (t, *J* = 6.7 Hz, 6H, CH_3_-6’ both isomers); 1.22–1.33 (m, 16H, CH_2_-2’, CH_2_-3’, CH_2_-4’, CH_2_-5’ both isomers); 1.34–1.48 (m, 2H, one of CH_2_-4 of **3a**, one of CH_2_-1’of **3a**); 1.50–1.58 (m, 2H, one of CH_2_-1’ of **2a**, one of CH_2_-1’ of **3a**); 1.66–1.75 (m, 2H, one of CH_2_-4 of **2a**, one of CH_2_-1’ of **2a**); 1.84 (m, 1H, one of CH_2_-3 of **3a**); 1.87–1.99 (m, 3H, CH_2_-3 of **2a**, one of CH_2_-4 of **2a**); 2.07 (m, 1H, one of CH_2_-3 of **3a**); 2.12 (m, 1H, one of CH_2_-4 of **3a**); 3.98 (m, 1H, H-5 of **2a**); 4.19 (dt, *J* = 13.1, 6.7 Hz, 1H, H-5 of **3a**); 5.45 (d, *J* = 4.5 Hz, 1H, H-2 of **2a**); 5.54 (dd, *J* = 5.0, 1.9 Hz, 1H, H-2 of **3a**); ^**13**^**C NMR** (150 MHz, CDCl_3_) δ: 14.09 (C-6’ of **2a**), 14.10 (C-6’ of **3a**), 22.61, 22.62, 26.09, 26.28, 29.16, 29.21, 31.82, 31.83 (C-2’, C-3’, C-4’, C-5’ both isomers), 29.35 (C-4 of **2a**), 29.40 (C-4 of **3a**), 33.12 (C-3 of **3a**), 34.09 (C-3 of **2a**), 35.66 (C-1’ of **3a**), 37.58 (C-1’ of **2a**), 78.52 (C-5 of **3a**), 81.21 (C-5 of **2a**), 98.30 (C-2 of **2a**), 98.47 (C-2 of **3a**); **IR** (film, cm^-1^): 3310 (s), 1459 (m), 1055 (w), 1007 (w).

Oxidation of (**±**)**-1b** (0.1 g) by HLADH, after 120 hours, gave a mixture of: (+)-(*R*)-**4b** (52%) and mixture of hemiacetals **2b** and **3b** (48%). The column chromatography of this mixture afforded 0.041 g (41% yield) of (+)-(*R*)-**4b**, ee = 33%, [α]_D_^20^ = + 5.6° (*c* 3.3, CHCl_3_) and 0.032 g (32%) of hemiacetals **2b** and **3b**.

Oxidation of (**±**)**-1b** (0.1 g) by HLADH recombinant from *E*. *coli*, after 120 hours, gave a mixture of: (+)-(*R*)-**4b** (31%) and mixture of hemiacetals **2b** and **3b** (28%). The column chromatography of this mixture afforded 0.025 g (25% yield) of (+)-(*R*)-**4b**, ee = 56%, [α]_D_^20^ = + 5.8° (*c* 2.6, CHCl_3_), 0.020 g (20%) of hemiacetals **2b** and **3b**.

### 6-Pentyltetrahydropiran-2-ols (mixture of isomers 2b and 3b)

Colorless liquid, ${\text{n}}_{\text{D}}^{25}$ = 1.4559; *cis* (**2b**) and *trans* (**3b**) isomers of the composition 68: 32%; ^**1**^**H NMR** (600 MHz, CDCl_3_) δ: 0.88 (t, *J* = 7.0 Hz, 6H, CH_3_-5’ both isomers); 1.11–1.90 (m, 28H, CH_2_-3, CH_2_-4, CH_2_-5, CH_2_-1’, CH_2_-2’, CH_2_-3’, CH_2_-4’ both isomers); 2.51 (s, 1H, OH of **3b**); 3.03 (d, *J* = 6.2 Hz, 1H, OH of **2b**); 3.40 (dddd, *J* = 9.1, 6.8, 4.9, 1.8 Hz, 1H, H-6 of **2b**); 3.92 (dddd, *J* = 11.3, 6.9, 5.1, 2.1 Hz, 1H, H-6 of **3b**); 4.69 (ddd, *J* = 8.7, 6.1, 1.9 Hz, 1H, H-2 of **2b**); 5.30 (m, 1H, H-2 of **3b**); ^**13**^**C NMR** (150 MHz, CDCl_3_) δ: 14.11 and 14.14 (C-5’ both isomers), 17.50, 22.16, 22.65, 25.16, 25.26, 29.73, 29.85, 30.45, 31.22, 31.92, 31.96, 32.96, 36.07, 36.21 (C-3, C-4, C-5, C-1’, C-2’, C-3’, C-4’ both isomers), 68.79 (C-5 of **3b**), 76.42 (C-5 of **2b**), 92.01 (C-2 of **3b**), 96.45 (C-2 of **2b**); **IR** (film, cm^-1^): 3403 (m), 1478 (m), 1043 (m).

### Extraction procedure using a Deryng apparatus

After the biotransformation, the combined aqueous fractions were placed in a 500 mL round flask and 0.1M NaOH was added portionwise to pH = 12. The aqueous fraction was heated for 2 hours, after the boiling point was reached. The vapours were condensed by means of a cold refrigerant. The main purpose of medium alkalization was conversion of prepared lactones **4a** and **4b**, by opening of lactone ring, into hydroxyacid salts, which are water-soluble and non-volatile compounds. After the extraction, the solvent (1 mL of cyclohexane) containing the volatile compounds—hemiacetals **2a**-**3a** and **2b**-**3b** was collected. Then, the reaction mixture was acidified by 0.1M HCl to pH = 3 and distilled again for 2 hours in Deryng apparatus. During the hydrodistillation of volatile compounds contained in the aqueous layer, γ- and δ-decalactones (**4a** and **4b**) were extracted with 1 mL of cyclohexane. The unreacted diol **1a** and **1b** remained in the aqueous medium, was used to next enzymatic oxidation. In this way, purified and separated substrate as well as volatile products without time-consuming and toxic solvent-free column chromatography were obtained.

### Oxidation of decane-1,4-diol (1a) and decane-1,5-diol (1b) isolated from products mixture after biotransformations with TEMPO/BAIB

To a stirred room temperature solution of diol **1a** and **1b** (0.03 g, 0.04 mmol), bis-acetoxyiodobenzene (BAIB, 0.014 g, 0.04 mmol) and (2,2,6,6-tetramethylpiperidin-1-yl)-oxyl (TEMPO, 0.001 g, 0.003 mmol) dissolved in methylene chloride (3 mL) were added sequentially. The reaction was conducted at room temperature. Progress of the reaction was monitored by TLC and GC. After 4 hours of the reaction, a saturated solution of Na_2_S_2_O_3_ (10 mL) was added and the mixture was extracted with diethyl ether (25 mL). The organic fraction was separated and washed with saturated NaHCO_3_ (10 mL) solution and water (10 mL). The combined aqueous layers were extracted three times with diethyl ether (25 mL). Ether extract was washed with brine, dried over anhydrous MgSO_4_, filtered and concentrated by rotary evaporation. After GC analysis with application of chiral column racemic mixture of γ-decalactone (**4a**) and δ-decalactone (**4b**) with insignificant enantiomeric excess (ee = 5%) were obtained.

### Oxidation of 5-hexyltetrahydrofuran-2-ols (mixture of isomers 2a and 3a) and 6-pentyltetrahydropiran-2-ols (mixture of isomers 2b and 3b) with pyridine dichromate

A mixture of diastereomeric hemiacetals **2a**, **3a** and **2b**, **3b** (0.025 g, 0.2 mmol) with pyridine dichromate (0.15 g, 0.4 mmol) and anhydrous sodium acetate (0.011 g, 0.1 mmol) dissolved in anhydrous methylene chloride (5 mL) were placed in a round bottom flask. The reaction was carried out at room temperature with continuous stirring. Progress of the reaction was monitored by TLC and GC analysis. After 4 hours of the reaction, solvent was evaporated, and the residue was extracted with hexane. The organic layer was filtered through Florisil, dried over anhydrous MgSO_4_ and evaporated in *vacuo* to afford enantiomerically enriched (–)-(*S*)-**4a** (ee = 24%) and (–)-(*S*)-**4b** (ee = 58%).

### Bioassays

#### Cultures of aphids and plants

Laboratory clone of peach-potato aphid *Myzus persicae* (Sulz.) was maintained on Chinese cabbage *Brassica pekinensis* in laboratory at 20°C, 65% of relative humidity, and L16:8D photoperiod. One to seven days old apterous females of *M*. *persicae* and 3 week old plants with 4–5 fully developed leaves were used for experiments. All experiments were carried out under the same conditions of temperature, relative humidity, and photoperiod. The bioassays were started at 10–11 a.m.

#### Behavioural Responses of Aphids

The effect of γ- and δ-decalactones (**4a** and **4b**) was assessed by monitoring settling behaviour of free individuals and aphid stylet activities in plant tissues (aphid probing and feeding behaviour).

#### Aphid settling

Aphids settle on a plant only when they accept it as a food source [29]. Therefore, the number of aphids that settle and feed on a given substrate is a good indicator of its suitability. This bioassay allows to study aphid host preferences under semi-natural conditions. Aphids are given free choice between control and treated leaves. The studied γ- and δ-decalactones (**4a** and **4b**) were applied by immersing a leaf in 0.1% ethanolic solution of a given compound for 30 sec. Control leaves of similar size were immersed in 70% ethanol that was used as a solvent for the tested lactones. Treated and control leaves were placed in a Petri dish and allowed to dry for 1 hour to permit the evaporation of the solvent. Next, aphids were placed in the dish along the line that divided the arena into two halves so that aphids could choose between treated (on one half of a Petri dish) and control leaves (on the other half of the dish). Aphids that settled, i.e. they did not move and the position of their antennae indicated feeding [30] on each leaf were counted at 1h, 2h, and 24h intervals after access to the leaf (8 replicates, 20 viviparous apterous females/replicate). Aphids that were moving or out of any of the leaves were not counted.

The data were analyzed using one way analysis of variance ANOVA (STATISTICA 6.1. package). If aphids showed clear preference for the leaf treated with the tested compound (P<0.05), the compound was described as having attractant properties. If aphids settled mainly on the control half of the leaf (P<0.05), the compound tested in the respective choice-test was stated a deterrent. From the data thus obtained the relative index of deterrence (D*I*) was calculated on the basis of formula *DI = C-T/C+T*, where *C* represents the number of aphids that settled on the control half of the leaf and *T* represents the number of aphids that settled on the half of the leaf treated using tested compound. The values of *DI* range between 1 (ideal deterrent) and –1 (ideal attractant). The compounds that modified aphid settling behaviour (i.e., the settling deterrents) were selected for further detailed study on probing behaviour.

#### Aphid probing and feeding behavior

Aphid probing and, especially, the phloem sap uptake by *M*. *persicae* was monitored using the technique of electronic registration of aphid probing in plant tissues, known as electrical penetration graph (EPG), that is frequently employed in insect–plant relationship studies considering insects with sucking-piercing mouthparts. In this experimental set-up, aphid and plant are made parts of an electric circuit, which is completed when the aphid inserts its stylets into the plant. Weak voltage is supplied in the circuit, and all changing electric properties are recorded as EPG waveforms that can be correlated with aphid activities and stylet position in plant tissues [31]. The parameters describing aphid behaviour during probing and feeding, such as total time of probing, proportion of phloem patterns E1 and E2, number of probes, etc., are good indicators of plant suitability or interference of probing by chemical or physical factors in individual plant tissues [32]. Lactones **4a** and **4b** were applied to one leaf of a 3-week old plant by immersing in 0.1% ethanolic solution of a given compound for 30 sec. Control leaves of similar size were immersed in 70% ethanol that was used as a solvent for the tested halogenated lactones. Treated and control leaves were allowed to dry for 1 hour before the start of the experiment to permit the evaporation of the solvent. Aphids were attached to a golden wire electrode with conductive silver paint and starved for 1 h prior to the experiment. Probing behaviour of 12 apterous females per studied lactone/aphid combination was monitored for 8 h continuously with a four-channel DC EPG recording equipment. Each aphid was given access to a freshly prepared leaf. Signals were saved on the computer and analysed using the PROBE 3.1 software provided by W. F. Tjallingii (www.epgsystems.eu) The following aphid behaviours were distinguished: no penetration (waveform 'np'–aphid stylets outside the plant), pathway phase-penetration of non-phloem tissues (waveforms 'ABC'), salivation into sieve elements (waveform 'E1'), ingestion of phloem sap (waveform 'E2'), and ingestion of xylem sap (waveform 'G'). The parametres derived from EPGs were analysed according to their frequency and duration in configuration related to activities in peripheral and vascular tissues.

One-way analysis of variance (ANOVA) was carried out on the experimental results. All calculations were performed using the STATISTICA 6.1 package (StatSoft, Tulsa, OK, USA).

## Results and Discussion

### Chemo-enzymatic synthesis

#### Enzymatic oxidation of racemic decane-1,4-diol (1a)

**Fig 1:**
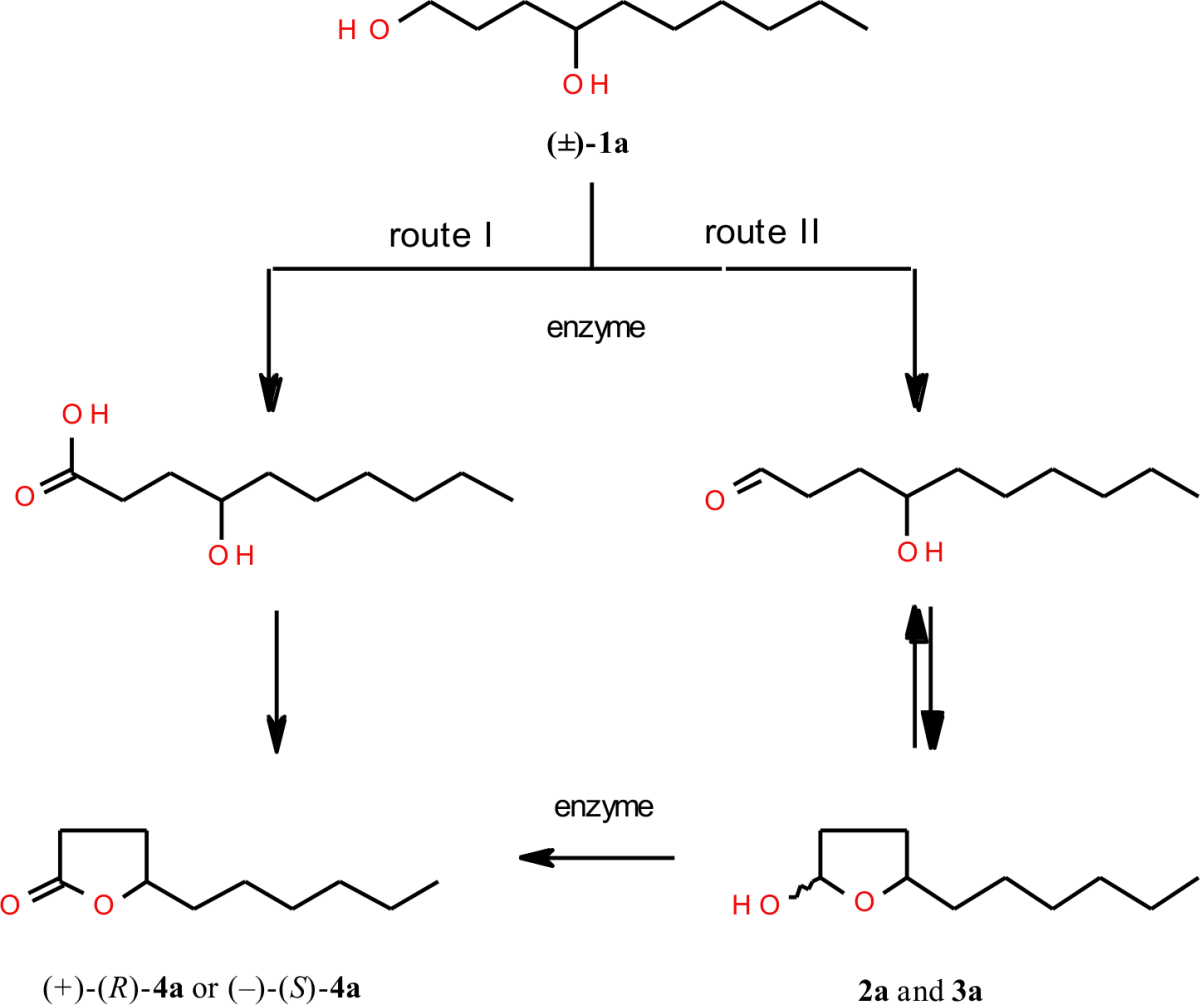
Two possible approaches of enzymatic oxidation of racemic decane-1,4-diol (1a).

There are two possible approaches to obtain lactone from decane-1,4-diol (**1a**) (Fig 1). In the first one, the primary hydroxyl group of diol is chemoselectively oxidized to carboxylic group. Then, in acidic pH, hydroxycarboxylic acid formed is cyclized to lactone product (route I). The second pathway assumes two steps of enzymatic oxidation of diol. Primary-secondary diol is initially oxidized to appropriate hydroxyaldehyde and then spontaneously formed hemiacetals are further oxidized to corresponding lactone (route II). As detected by GC and confirmed by spectroscopic methods, the products of the first step of oxidation of decane-1,4-diol (**1a**), instead of the hydroxyaldehyde, were their corresponding hemiacetals **2a** and **3a**.

Due to the fact that HLADH isolated directly from horse liver is not commercially available now, we used also the enzyme HLADH recombinant in *Escherichia coli*. To extend our investigations, in looking for the most effective biocatalyst, we applied several other alcohol dehydrogenases in oxidation processes of decane-1,4-diol (**1a**) (Table 1). It was interesting to evaluate the ability of different alcohol dehydrogenases and compare their stereoselectivity in transformation of such substrate.

**Table 1:** The composition (in % according to GC) of products mixture after 5 days of enzymatic oxidation of racemic decane-1,4-diol (1a).

Enzyme	OXIDATION CONDITIONS								Substrate	Products		
	Coenzyme		Temperature[°C]			pH			1a	2a and 3a	4a	
	NAD^+^/ NADP^+^	FMN	25	35	45	7.2	8.5	9.0	[%]	[%]	[%]	*ee* [%]
HLADH	**√**	**√**	**√**					**√**	**4**	**4**	**92**	***22* (–)-(*S*)**
HLADH*	**√**	**√**	**√**					**√**	nd	nd	100	*0*
	**√**	**√**		**√**			**√**		nd	nd	100	*0*
	**√**	**√**	**√**			**√**			83	nd	17	*20* (–)-(*S*)
	**√**		**√**					**√**	32	1	**67**	***22* (–)-(*S*)**
BSADH	**√**		**√**					**√**	89	2	9	*-*
	**√**	**√**			**√**			**√**	98	nd	2	*-*
LKADH	**√**	**√**	**√**			**√**			100	nd	nd	
	**√**	**√**	**√**				**√**		95	1	4	*-*
PADH I	**√**	**√**	**√**			**√**			60	nd	40	*26* (–)-(*S*)
	**√**	**√**	**√**				**√**		100	nd	nd	
PADH II	**√**	**√**	**√**			**√**			91	8	1	*-*
	**√**	**√**	**√**				**√**		62	2	36	*18* (–)-(*S*)
PADH III	**√**	**√**	**√**			**√**			59	18	23	*53* (+)-(*R*)
	**√**	**√**	**√**				**√**		**nd**	**41**	**59**	***48* (+)-(*R*)**
YADH	**√**	**√**	**√**			**√**			90	4	6	*10* (+)-(*R*)
	**√**	**√**	**√**				**√**		88	nd	12	*8* (+)-(*R*)

*alcohol dehydrogenase recombinant from *E*. *coli* (HLADH); *ee*-enantiomeric excess; nd-not detected

Most of the biocatalysts used, except LKADH and BSADH, transformed decane-1,4-diol (**1a**) to one of the enantiomerically enriched isomers of γ-decalactone (**4a**). Oxidation of (**±**)**-1a** catalyzed by both forms of HLADH, PADH I and PADH II gave (–)-(*S*)-isomer of **4a** with ee = 18–30%. In contrary to this, transformations with PADH III and YADH led to product with predominance of (+)-(R) enantiomer (ee = 8–53%). In most transformations of (**±**)**-1a** a mixture of hemiacetals **2a** and **3a** were identified by GC analysis in small amounts (1–8%). Taking into consideration the results of preliminary screening, the preparative transformations of the diol **1a** were carried out to obtain both enantiomerically enriched isomers of γ-decalactone. Enzyme HLADH catalyzed the oxidation of diol **1a** to the (–)-(*S*)-**4a** with ee = 20% ([α]_D_^25^ = – 5.8° (*c* 1.5, CHCl_3_)), while PADH III-mediated oxidation gave (+)-(*R*)-**4a** with significantly higher enantiomeric excess (ee = 80%) (Table 2). Differences in enantiomeric excess of (+)-(*R*)-**4a** isomer of γ-decalactone between screening (ee = 48%, Table 1) and preparative scale (ee = 80%, Table 2) experiments catalyzed by PADH III, are due to different diol **1a** conversion, in screening scale 59% and in preparative scale 16% respectively. It is frequent observation in biotransformation that the higher conversion, the smaller enantiomeric excess of lactone product.

**Table 2:** The composition (in % according to GC) of products mixture in the course of preparative oxidation of racemic decane-1,4-diol (1a) catalyzed by alcohol dehydrogenases.

Enzyme	Time	Substrate		Products			
		1a		2a and 3a		4a	
	[hours]	[%]	*ee* [%]	[%]	*de* [%]	[%]	*ee* [%]
HLADH	24	34	*-*	11	*-*	55	*22* (–)-(*S*)
	48	nd		21	*-*	79	*20* (–)-(*S*)
PADH III	48	84	*-*	16	*-*	nd	
	120	66	*0*	18	*10* (**2a**)	16	*80* (+)-(*R*)

*ee*-enantiomeric excess, *de*-diastereoisomeric excess; nd-not detected

#### Chemical oxidation of diol 1a and hemiacetals 2a and 3a

Enantiomerically enriched mixture of hemiacetals **2a** and **3a**, prepared as a result of preparative transformation catalyzed by PADH III, was oxidized with pyridine dichromate, in order to afford reverse isomer of lactone product **4a** formed and determine its enantiomeric excess. As it turned out, the enantiomeric excess of (–)-(*S*)-**4a** formed was ee = 24%. In next experiment, the isolated unreacted diol **1a**, from the same biotransformation, was oxidized with TEMPO/BAIB to racemic mixture of lactone **4a**. Studies of stereoselectivity of biotransformation of decane-1,4-diol (**1a**) are consistent with our results recently published concerning oxidation of *erythro*- and *threo*-3-methyoctane-1,4-diols to corresponding *cis*- and *trans*-whisky lactones [27].

**Fig 2:**
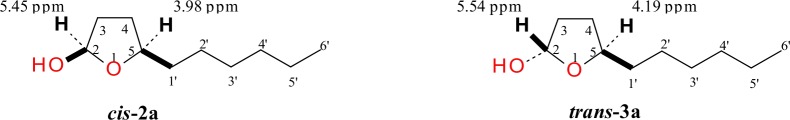
Space structures and chemical shifts of signals from protons H-2 and H-5 in hemiacetals 2a and 3a.

Hemiacetals **2a** and **3a**, as a diastereoisomeric *cis*/*trans* mixture, were isolated by column chromatography from products mixture after 120 hours of oxidation catalyzed by PADH III. Identification of their structures were made on the basis of chemical shifts of the signals from proton H-5 in the ^1^H NMR spectrum. Considering the space structure of individual diastereoisomers **2a** and **3a**, the position of hydroxyl group and hexyl group on the same side of the lactol ring will point to the *cis* isomer of hemiacetal (**2a**) (Fig 2). However, in the *trans* isomer (**3a**) both substituents are located in the opposite sides of the lactol ring. The ratio of *cis*/*trans* isomers (45% of **2a** and 55% of **3a**) was determined from the integration of signals of proton H-5. Doublet of triplets from the proton H-5 in the ^1^H NMR spectrum of the *trans* isomer **3a** is shifted downfield (δ = 4.19), in comparison with its location in the ^1^H NMR spectrum of the *cis* isomer **2a** (δ = 3.98). Such a difference in chemical shift is due to deshielding effect of the oxygen atom of the hydroxyl group located on the same side of hemiacetal ring as the proton H-5 in the *trans*-isomer **3a**. The presence of one-proton multiplets from H-2 respectively at 5.45 (for **2a**) and 5.54 ppm (for **3a**) in the ^1^H NMR spectrum indicates the presence of hemiacetal ring in the molecule.

#### Enzymatic oxidation of racemic decane-1,5-diol (1b)

**Fig 3:**
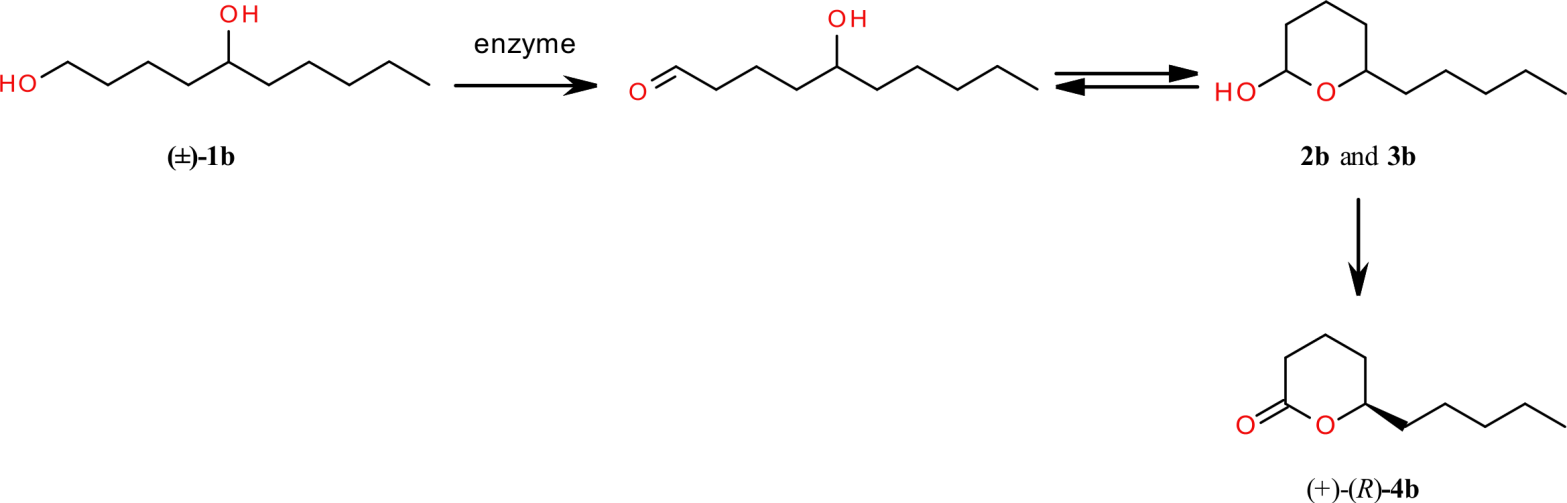
Enzymatic oxidation of racemic decane-1,5-diol (1b).

On the basis of our previous results from HLADH-catalyzed oxidation of various 1,5-diols to corresponding δ-lactones [26] we expected high yield of transformation as well as stereoselectivity in formation of optically active δ-decalactone (**4b**). Likewise in oxidation of 1,4-decadiol (**1a**), the formation of δ-decalactone (**4b**) is conducted *via* two steps of oxidation (Fig 3).

Among tested alcohol dehydrogenases, HLADH isolated from horse liver as well as recombinant in *Escherichia coli*, were only ones effective biocatalysts tried in the oxidation of decane-1,5-diol (**1b**) to δ-decalactone (**4b**) (Table 3). Depending on the oxidation conditions, optically active (+)-(*R*)-δ-decalactone (**4b**) with enantiomeric excess in the range 30–50%, together with mixture of hemiacetals **2b** and **3b**, were formed.

**Table 3:** The composition (in % according to GC) of products mixture after 5 days of enzymatic oxidation of racemic decane-1,5-diol (1b).

Enzyme	OXIDATION CONDITIONS								Substrate	Products		
	Coenzyme		Temperature[°C]			pH			1b	2b and 3b	4b	
	NAD^+^/ NADP^+^	FMN	25	35	45	7.2	8.5	9.0	[%]	[%]	[%]	*ee* [%]
HLADH	**√**	**√**	**√**					**√**	**9**	**41**	**50**	***46* (+)-(*R*)**
HLADH*	**√**	**√**	**√**					**√**	23	36	41	*30* (+)-(*R*)
	**√**	**√**		**√**			**√**		30	49	21	*38* (+)-(*R*)
	**√**	**√**	**√**			**√**			**58**	**17**	**25**	***50* (+)-(*R*)**
	**√**		**√**					**√**	62	29	9	*48* (+)-(*R*)
BSADH	**√**		**√**					**√**	68	29	3	*-*
	**√**	**√**			**√**			**√**	98	2	nd	
LKADH	**√**	**√**	**√**			**√**			100	nd	nd	
	**√**	**√**	**√**				**√**		100	nd	nd	
PADH I	**√**	**√**	**√**			**√**			90	10	nd	
	**√**	**√**	**√**				**√**		100	nd	nd	
PADH II	**√**	**√**	**√**			**√**			83	9	8	*-*
	**√**	**√**	**√**				**√**		81	14	5	*-*
PADH III	**√**	**√**	**√**			**√**			77	23	nd	
	**√**	**√**	**√**				**√**		68	32	nd	
YADH	**√**	**√**	**√**			**√**			100	nd	nd	
	**√**	**√**	**√**				**√**		100	nd	nd	

*alcohol dehydrogenase recombinant from *E*. *coli* (HLADH); *ee*-enantiomeric excess; nd-not detected

Based on screening scale experiments important to notice is fact that, in contrary to formation of γ-lactone ring, all tested alcohol dehydrogenases, with exception of HLADH, were poor or inadequate biocatalysts to afford δ-lactone product (Table 3). It confirms that HLADH in oxidation process of decane-1,5-diol (**1b**) is irreplaceable enzyme. In preparative scale (+)-(*R*)-δ-decalactone (**4b**) with higher optical purity (ee = 56%) was achieved after five days of biotransformation catalyzed by HLADH recombinant in *Escherichia coli* (Table 4).

**Table 4:** The composition (in % according to GC) of products mixture in the course of preparative oxidation of racemic decane-1,5-diol (1b) catalyzed by alcohol dehydrogenases.

Enzyme	Time	Substrate		Products			
		1b		2b and 3b		4b	
	[hours]	[%]	*ee* [%]	[%]	*de* [%]	[%]	*ee* [%]
HLADH	48	25	-	32	-	43	*37* (+)-(*R*)
	120	nd		48	36 (**2b**)	52	*33* (+)-(*R*)
HLADH*	48	89	-	11	-	nd	
	120	41	*5*	28	58 (**2b**)	31	*56* (+)-(*R*)

*alcohol dehydrogenase recombinant from *E*. *coli* (HLADH); *ee*-enantiomeric excess

*de*-diastereoisomeric excess; nd-not detected

**Fig 4:**
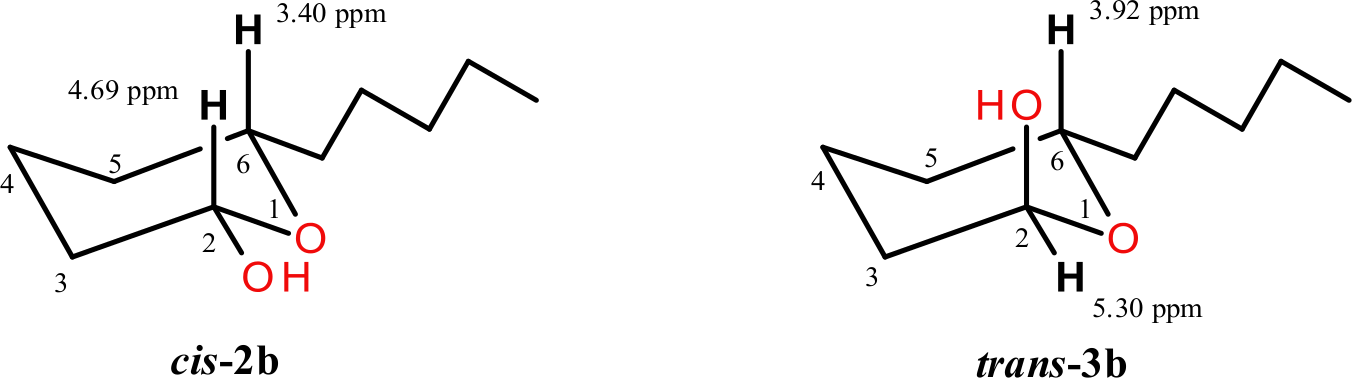
Space structures and chemical shifts of signals from protons H-2 and H-6 in hemiacetals 2b and 3b.

Through spectroscopic analysis (^1^H NMR, ^13^C NMR, HMQC, COSY) the products of the first step of oxidation process were determined as diastereoisomeric *cis*/*trans* mixture of hemiacetals **2b** and **3b**. Identification of these products were made on the basis of chemical shifts of the signals from protons H-2 and H-6 in the ^1^H NMR spectrum. The signals of these protons were assigned to the corresponding isomers on the basis of their chemical shifts. Assuming the chair conformation and equatorial position of alkyl group at C-6 (Fig 4) in the *cis* isomer hydroxyl group was also located in equatorial position, whereas in *trans* isomer it occupied axial position. In *trans* isomer the multiplet of H-6 is shifted downfield (δ = 3.92) by the axial hydroxyl group, in comparison with its location in the ^1^H NMR spectrum of the *cis* isomer (δ = 3.40). On the other hand, the signal of equatorial proton H-2 in the *trans* isomer is shifted downfield (δ = 5.30) by deshielding effect of neighboring oxygen atom, in comparison with the chemical shift (δ = 4.69) of the axial proton H-2 found in the spectrum of the *cis* isomer. The ratio of *cis*/*trans* isomers (68% of **2b** and 38% of **3b**) was determined from the integration of multiplets of protons H-6 and H-2.

#### Chemical oxidation of diol 1b and hemiacetals 2b and 3b

As we plan to obtain both enantiomers of δ-decalactone (**4b**) we performed chemical oxidation of corresponding unreacted isomers of lactols **2b**, **3b** and diol **1b**. The diastereoisomeric mixtures of **2b** and **3b**, isolated from both preparative transformations, were oxidized with pyridine dichromate to opposite isomer of δ-decalactone (**4b**). In this way enantiomerically enriched (–)-(*S*)-isomer (**4b**) with ee = 32% ([α]_D_^25^ = –5.2° (*c* 3.3, CHCl_3_)) and ee = 58% ([α]_D_^25^ = –13.7° (*c* 3.6, CHCl_3_)) was obtained as a product of two-step chemoenzymatic synthesis. Firstly, oxidation of diol **1b** to lactol **2b** and **3b** catalyzed by HLADH isolated from horse liver as well as recombinant in *Escherichia coli*, followed by chemical oxidation took place. On the other hand the unreacted diol **1b** from biotransformation was subjected to the oxidation with TEMPO/BAIB. The process delivered (–)-(*S*)-δ-decalactone (**4b**) with insignificant enantiomeric excess (ee = 5%).

Our goal of chemical oxidation of unreacted diol **1b** and diastereoisomers of hemiacetals **2b**, **3b** formed during biotransformation was to evaluate the enantioselective step in enzymatic oxidation of primary-secondary 1,5-diols. Similarly to biotransformations of 1,4-diols, in oxidation of 1,5-diols the second stage of diol oxidation—oxidation of the corresponding hemiacetals to lactone—determines the enantioselectivity of the whole oxidation process. To our knowledge stereoselective enzymatic oxidation of diols to lactones is novel and effective method to obtain diastereomerically enriched hemiacetals, which can be further chemically oxidized to opposite lactone isomer.

### Biological activity

#### Aphid settling

**Fig 5:**
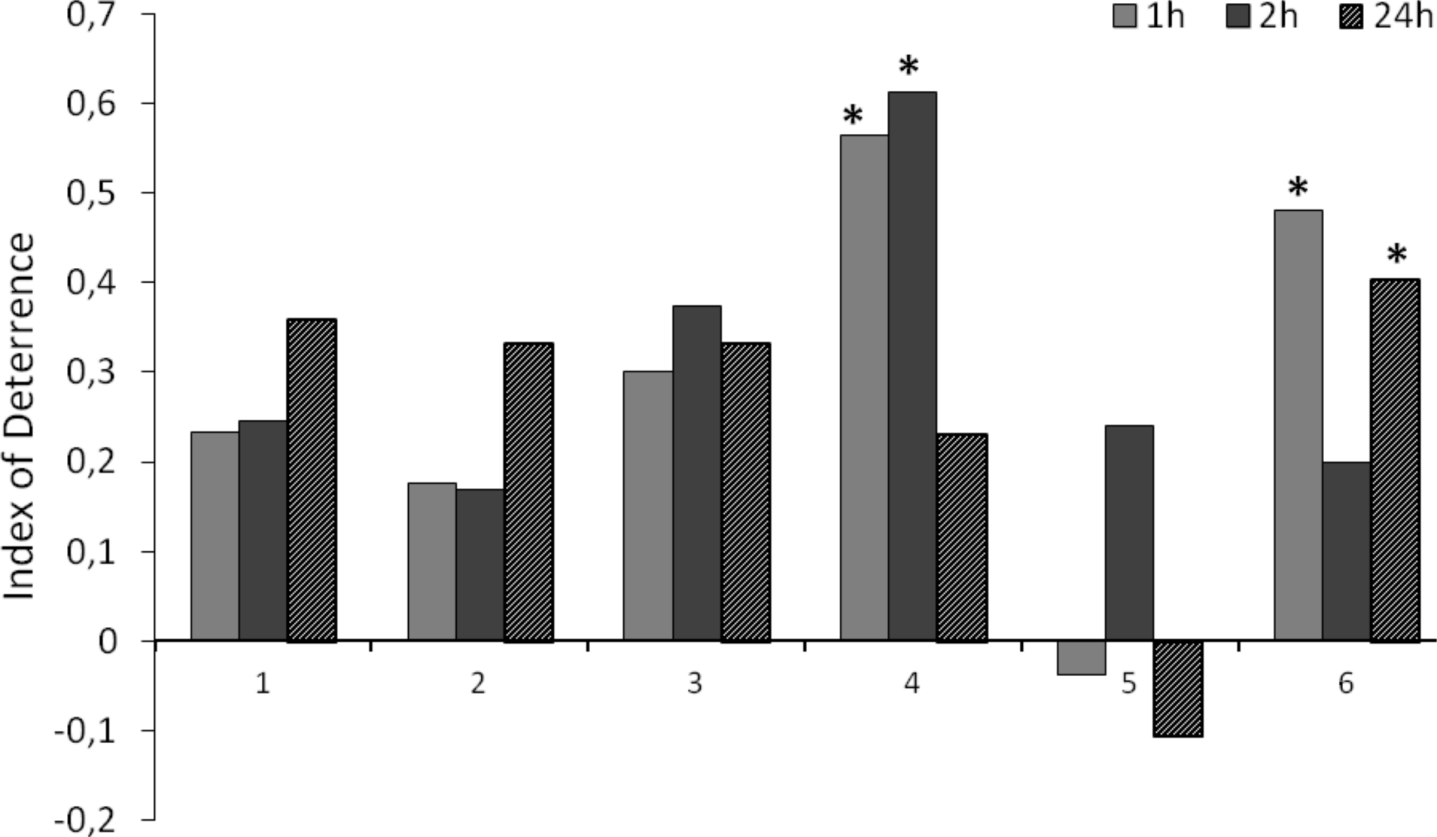
Effect of decalactones and their pure enantiomers (1–6) on the settling preferences of *Myzus persicae* in the choice test. 1—(±)-δ-decalactone (**4b**), 2—(+)-(*R*)-δ-decalactone (**4b**), 3—(–)-(*S*)-δ-decalactone (**4b**), 4—(±)-γ-decalactone (**4a**), 5—(+)-(*R*)-γ-decalactone (**4a**), 6—(–)-(*S*)-γ-decalactone (**4a**). Data are expressed as values of indices of deterrence (*DI*). * *P <* 0.05 (Student *t*-test).

The behavior of *Myzus persicae* during settling on leaves varied depending on the decalactone applied (Fig 5). Racemic γ-decalactone (**4a**) showed a relatively high significant deterrent activity as soon as one hour after application (index of deterrence ID_1_ = 0.6), but the deterrent effect ceased in the course of time (ID_24_ = 0.2). However, considering the enantiomeric composition of γ-decalactone (**4a**), (–)-(*S*)-γ-decalactone discouraged aphids to settle on treated leaves significantly at least 24 hours that followed the application (ID_24_ = 0.4), while (+)-(*R*)-γ-decalactone showed slight, but not statistically significant attractant properties (ID_24_ = –0.1). Different response of aphids to pure enantiomers was probably the reason of the short-lived deterrent activity of the γ-decalactone (**4a**) racemate reported earlier. Neither the racemic δ-decalactone (**4b**) nor any of its enantiomers appeared to alter the host acceptance behavior by *M*. *persicae*.

#### Aphid probing

The EPG recording revealed all kinds of aphid activities related to plant penetration: non-probing, pathway phase ('C') including the unidentified ('derailed') stylet movements 'F', phloem salivation and sap ingestion 'E1' and 'E2', respectively, and xylem sap uptake 'G'. 'F' and 'G' activities occurred sporadically irrespective of a treatment and were included in pathway phase for statistical analysis.

**Fig 6:**
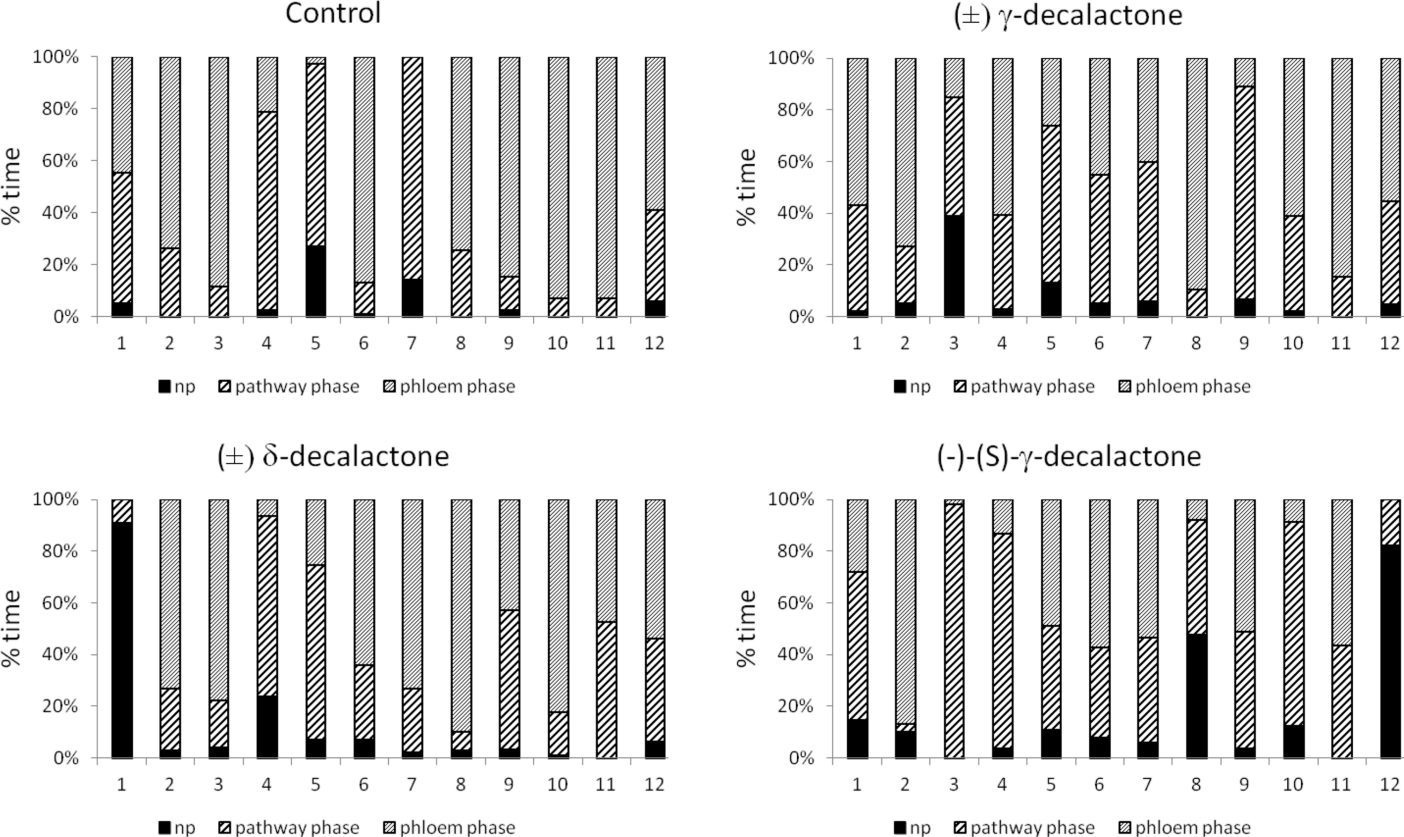
Analysis of *Myzus persicae* probing activities on plants after application of decalactones, expressed as the proportion of behavioural events in individual aphids (numbers on X axis represent individual aphids). np–no probing, pathway phase–probing in parenchymatous tissues, phloem phase–salivation and sap ingestion of phloem sap.

**Fig 7:**
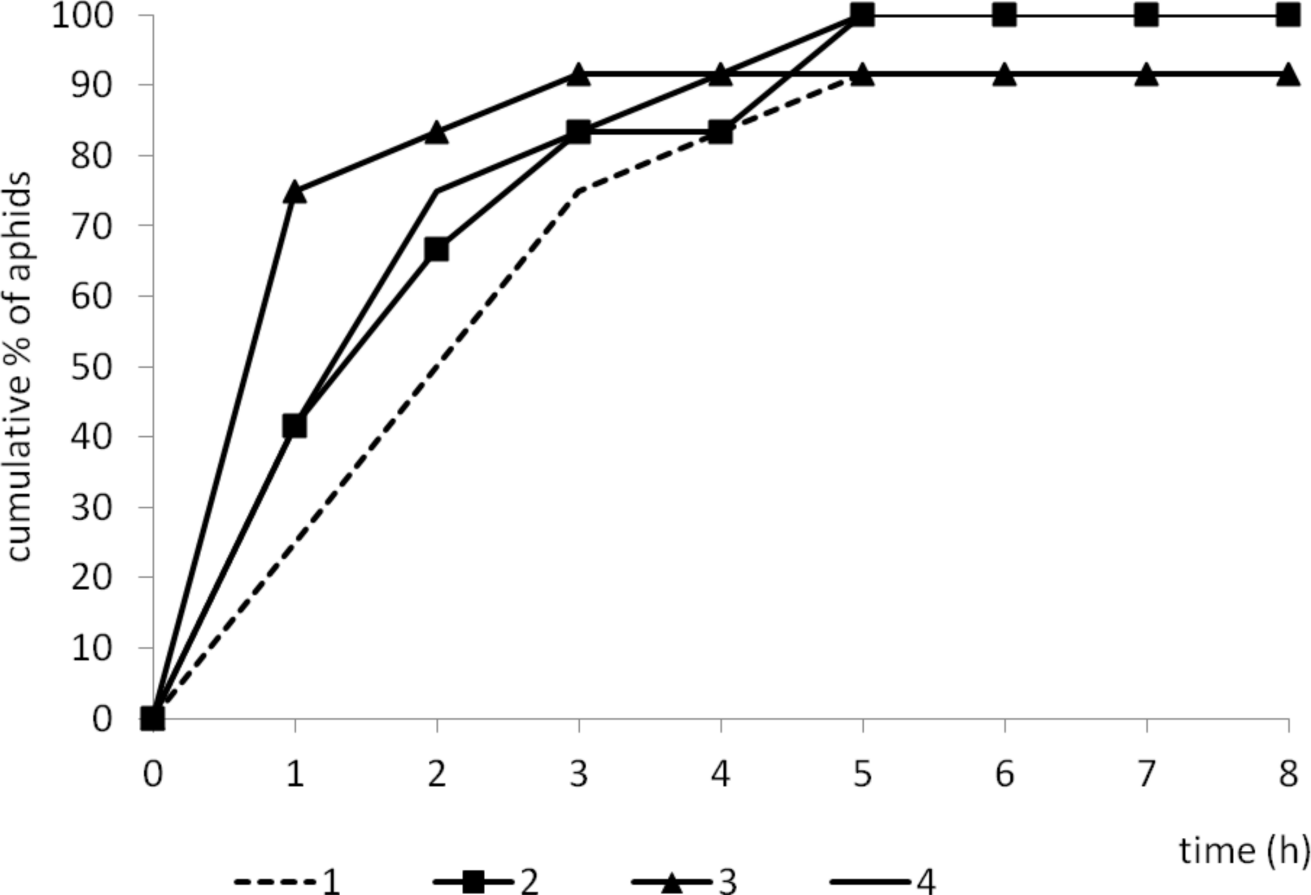
Cumulative proportion of *Myzus persicae* that showed probing activities in sieve elements, phloem salivation (E1) and sap ingestion (E2) in the course of the 8 hours after application of decalactones. 1—control, 2—(±)-δ-decalactone (**4b**), 3—(±)-γ-decalactone (**4a**), 4—(–)-(*S*)-γ-decalactone (**4a**).

**Fig 8:**
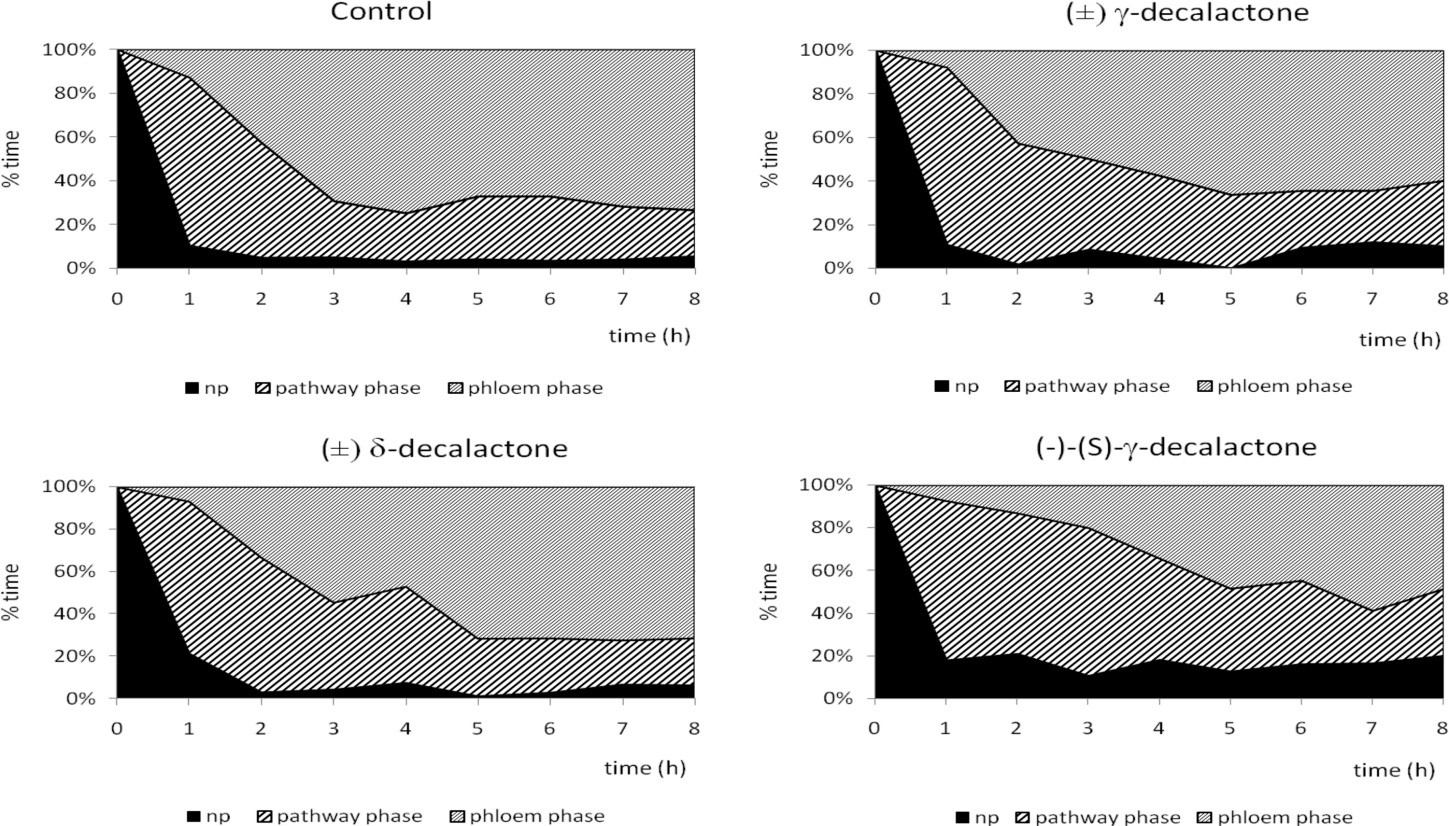
Trends in *Myzus persicae* probing behavior activities in the course of the 8 hours after application of decalactones.

Despite individual variation within experimental groups (Fig 6), certain trends in aphid behavior could be observed. On control plants, aphid probing was rarely interrupted: there were twelve probes of 1.5 hour duration per aphid, on average during eight hours of continuous EPG recording. Consequently, plant penetration occupied 95% of experimental time and ca. 70% of that time was phloem phase that included 83% activities associated with sap ingestion. Sap ingestion periods were four hours long, on average, and more than 90% of aphids reached sieve elements within the 8-hour experiment, generally in 20 minutes within a successful probe. Phloem phase prevailed among probing activities from the second hour of the experiment onwards in the majority of aphids (Table 5, Figs 6–8). On racemic δ-decalactone (**4b**)-treated plants, probing activities occupied ca. 93% of experimental time, 57% of probing was dedicated to phloem phase, and 41% to sap ingestion which was slightly, but not significantly, less than in aphids on control plants. There were ca. 16 probes per aphid, their mean duration was approximately less than one hour, and an average period of phloem phase was nearly two hours. Sap ingestion periods were 2.4 hour long on average. All aphids reached sieve elements (Fig 7) and started ingestion usually in 20 minutes within a successful probe (Table 5). Moreover, phloem phase was the main aphid activity of nearly all experimental aphids on treated leaves (Fig 6), and it predominated over other activities from the second hour of the experiment onwards (Fig 8). In respect to the application of racemic γ-decalactone (**4a**), there was no significant decrease in the total duration of plant tissues penetration by aphids in comparison to control. However, aphid behaviour during probing in phloem vessels was altered: although the total duration of phloem phase was similar to that on control plants, the individual periods of sap ingestion were significantly shorter. The average phloem phase was 2.3 times shorter and mean duration of sustained ingestion was 3.5 times lower in aphids on treated plants than on control. Altogether, the total duration of sap ingestion during the 8-hour experiment was two times lower as compared to control although all aphids reached sieve elements (Table 5, Fig 6). Pathway activities (i.e., probing in non-phloem tissues) was a predominant activity during three successive hours after the onset of the experiment (Figs 6 and 8). Aphids on (–)-(*S*)-γ-decalactone (**4a**)-treated leaves were not prevented from probing but their activities were limited mainly to penetration in non-phloem tissues. Total and average durations of phloem phase were 1.7 and 6.0 times shorter than on control leaves, respectively. Moreover, activities within phloem phase associated with sap ingestion were also constrained: the proportion of sap ingestion within phloem phase was 32%, while on control leaves 83% (Table 5). Pathway activities predominated during six hours of the 8-hour experiment in the majority of aphids and there were individuals (8% of the experimental group) that did not reach phloem phase during that time (Figs 6 and 7 and 8).

**Table 5:** Alteration of *Myzus persicae* behaviour during probing after exposure to decalactones^a^.

PARAMETERS		control	(±)-δ-decalactone (4b)	(±)-γ-decalactone (4a)	(–)-(S)-γ-decalactone (4a)
NON-SEQUENTIAL PARAMETERS					
Total duration of nonprobing	min	25.0 ±11.1	31.5 ±10.1	35.0 ±14.6	79.4 ±33.5
No. of probes	#	12.6 ±4.1	15.8 ±4.1	12.7 ±2.8	22.7 ±6.9
No. of E1+E2 phloem phases	#	1.8 ±0.582	3.4 ±0.5*	3.0 ±0.5*	4.7 ±0.8*
Total duration of the phloem phase E1+E2	min	287.0 ±48.4	257.0 ±39.5	239.3 ±35.4	164.8 ±39.1
Average duration of phloem phase E1+E2	min	245.1 ±47.9	117.4 ±30.6	108.0 ±25.0*	41.2 ±12.6*
Total duration of the phloem phase E2	min	240.2 ±49.0	126.3 ±36.7	137.4 ±26.7	89.7 ±31.3*
Average duration of the phloem phase E2	min	209.3 ±51.4	58.8 ±32.3*	21.5 ±8.6*	3.9 ±1.3*
Total duration of sustained ingestion E2	min	238.2 ±49.0	106.5 ±37.2	112.8 ±27.3*	52.5 ±24.9*
Average duration of sustained ingestion E2	min	259.3 ±52.4	141.8 ±49.9	74.8 ±29.5*	30.4 ±10.0*
SEQUENTIAL PARAMETERS
Time to the first probe (in recording; = d_1Np)	min	1.3 ±0.5	1.2 ±0.6	3.6 ±3.4	0.5 ±0.3*
Duration of the first probe	min	23.0 ±10.4	12.8 ±3.9	44.2 ±17.2	54.4 ±39.3
Time to the first phloem phase E from the first probe	min	136.7 ±37.6	97.8 ±24.9	88.4 ±24.2	79.7 ±38.3
Time to the first E within the first probe with E	min	16.7 ±2.3	18.8 ±3.5	20.7 ±5.1	12.5 ±3.5
Average time to the first E within probes	min	19.3 ±2.4	22.4 ±2.4	21.6 ±3.1	13.6 ±3.3
Minimum time to the first E within probes	min	18.6 ±2.5	17.5 ±3.0	15.0 ±3.2	8.6 ±3.8*
No. of brief probes < 3 min before first E	#	3.2 ±1.3	3.7 ±1.3	1.2 ±0.6	1.5 ±0.6

^a^Values represent the means of n = 12 replicates ±SE.

An asterisk within a row denotes statistically significant differences in relation to control (P<0.05).

EPG-recorded signals provide an insight into the cryptic mode of aphid-plant interactions during probing. Due to the specific structure of sucking-piercing mouthparts, direct observation of aphid behaviour associated with feeding is impossible. Therefore, parameters derived from EPG experiments are good indicators of plant suitability for aphids. Additionally, the interpretation of the results shows the tissular localization of deterrent (or attractant) factors and, consequently, exposes the physiological effects in aphids. For example, long penetration time of non-phloem tissues as compared to total penetration time, high number of short vs. long probes before the first phloem phase, relatively long time to 1^st^ phloem phase within a probe, and a failure in finding sieve elements may be interpreted as preingestional effects of antifeedants that restrain aphid probing at the level of non-phloem tissues. Short (<10 min) probes are limited either to epidermis (<2 min.) or do not reach beyond mesophyll (2–10 min.). Similarly, the short total and mean durations of phloem sap ingestion and high proportion of salivation during penetration of phloem vessels may point to the ingestional level of feeding deterrence [32, 33]. On suitable host plants, the sap ingestion periods may last for many hours with no interruption [34, 35]. Supplementary to EPG experiments is the choice-test on aphid settling, which was also used in this work. In the case of compounds that are inactive at pre- and (or) ingestional levels, it can reveal postingestive activity if the compound deters aphid settling at least 24 hours after exposure. In our study, the application of δ-decalactone (**4b**), irrespective of its enantiomeric purity did not hinder aphid settling on leaves and it was confirmed in the EPG study. Aphid probes were relatively long and non-probing periods were relatively short and comparable to those on control plants. Likewise, the sap ingestion phases were relatively long and uninterrupted. In contrast, racemic γ-decalactone (**4a**) caused a negative response of aphids during settling. However, the effect was short-lived, which was further explained in the EPG study. It appeared that the enantiomeric purity was crucial in the expression and the durability of the deterrent properties. Only (–)-(*S*)-γ-decalactone (**4a**) had strong and durable (i.e. lasting for at least 24 hours) limiting effect, expressed at phloem level. Therefore, this compound (–)-**4a** can be considered an ingestive deterrent.

In summary, the deterrent activity of decalactones (**4a** and **4b**) against aphids depended on the size of the lactone ring and the enantiomeric purity of the compounds. The decalactone (**4b**) containing 6-membered ring appeared inactive against *M*. *persicae* while the decalactone (**4a**) with 5-membered ring deterred aphid feeding at ingestional level. Only the levorotatory *S* isomer of **4a** was active.
